# Outcomes of dental implants in young patients with congenital versus non-congenital missing teeth

**DOI:** 10.1186/s40729-021-00362-7

**Published:** 2021-08-23

**Authors:** Yousef Al Najam, Ali Tahmaseb, Dorothee Wiryasaputra, Eppo Wolvius, Brunilda Dhamo

**Affiliations:** Department of Oral and Maxillofacial Surgery, Erasmus University Medical Center Rotterdam, PO Box 2040, CA 3000 Rotterdam, the Netherlands

**Keywords:** Tooth, Agenesis, Non-congenital, Implant, Success, Survival

## Abstract

**Objective:**

This cross-sectional study aims to investigate the effect of the cause of missing teeth on the *survival* and subjective *success* of dental implant treatment (DIT) in young patients with missing teeth due to non-congenital causes (tooth loss) in comparison to patients with missing teeth because of congenital causes (hypodontia and oligodontia).

**Material and methods:**

All patients were asked 7 questions to extract information about the survival and subjective success of DIT. Implant survival function was designed using the Kaplan-Meier analysis. Differences in implant success outcomes were studied using binary logistic regression analysis.

**Results:**

One hundred ten patients aged 18 to 40 years old were included, whereof 32 patients with tooth loss, 25 patients with hypodontia and 53 patients with oligodontia. In the tooth loss group, implant survival reached 96.9%; in the hypodontia group 96.0%; and in the oligodontia group 88.7%. Regarding subjective implant success, patient satisfaction was significantly higher (*p < 0.040*) among patients with congenital missing teeth in comparison to patients with tooth loss. Other implant success components showed no statistically significant difference (*p* > 0.050) between the groups.

**Conclusion:**

The cause of missing teeth does not influence implant survival. However, the cause of missing teeth does have a significant impact on patient satisfaction (implant success), ascertaining young patients with congenital missing teeth as more satisfied of DIT than young patients with tooth loss.

**Clinical relevance:**

Young patients with tooth agenesis and with an increased number of missing teeth are more content about the treatment with dental implants than patients with tooth loss. Furthermore, a consensus regarding the assessment of implant success is an essential concern for clarification.

**Supplementary Information:**

The online version contains supplementary material available at 10.1186/s40729-021-00362-7.

## Introduction

Tooth agenesis is defined as the congenital absence of one or more missing teeth excluding the third molars [37]. Tooth agenesis is the most common congenital dental abnormality with a prevalence that varies between 0.15% and 16.2% in different study populations [32]. Based on the number of missing teeth, tooth agenesis is classified in hypodontia (1–5 missing teeth), oligodontia (6–27 missing teeth), and anodontia (28 missing teeth) [36]. Non-congenitally absence of teeth, known as tooth loss, can be presented as partial or total edentulism. Edentulism is a major public health problem affecting 6–80% of the people in different countries [12, 22]. Tooth loss occurs as a result of severe dental caries, trauma, and pathogenic mobility due to severe periodontitis [33]. In order to bring back the functionality and aesthetics of the dentition to the patients with missing teeth, a multidisciplinary team of dentists, orthodontists, maxillofacial surgeons, and psychologists is needed [14].

Dental implant treatment (DIT) is recognized as the most successful treatment for missing teeth because of the high survival rate of more than 94.0% over a mean period of 13 years [16, 29]. However, several factors such as smoking, unhealthy/sugary diet, bruxism, xerostomia, osteoporosis, diabetes, and radiotherapy can contribute to unsatisfactory outcomes and very early failure of the implants in patients with missing teeth due to congenital or non-congenital causes [34]. A systematic review of the literature has shown that the mean implant survival in patients with oligodontia is 93.7%, varying from 35.7 to 98.7% over a period of 18 years [14]. The variance of 35.7 to 98.7% is explained by the inclusion of studies performed in children (< 15 years old) with lower bone quantity and thus a higher risk for DIT failure rate. In another study, patients with hypodontia and oligodontia showed high satisfaction and masticatory function (69.4%), high phonetic ability (80.6%), and high implant success (88.4%) according to Albrektsson criteria [3].

Studies about DIT in patients with missing teeth due to non-congenital causes such as severe periodontitis have reported higher survival rates between 97% and 100% in short-term follow-up period of 2 to 8 years [7, 23, 25, 26]. Long-term follow-up investigations have resulted in survival rates between 83% and 96% after 10 years [7, 20, 21, 23–26]. Accordingly, the follow-up time is an important determinant for the DIT survival. Another study which examined the implant success in patients with tooth loss has reported success rates between 38.5% and 77.9% over a period of 3 to 6 years [27].

Based on the reported values, outcomes of DIT in patients with congenital missing teeth might be less favorable compared to outcomes in a group of patients with tooth loss due to caries, infection, or trauma. However, due to a lack of comparative studies, the question whether the outcomes of DIT in patients with congenital missing teeth differ from patients with tooth loss remains unraveled. Therefore, the aim of this research is to study the effect of the cause of missing teeth on the *survival* and subjective *success* of DIT in patients with missing teeth due to non-congenital causes (tooth loss) in comparison to patients missing teeth because of congenital causes (hypodontia and oligodontia).

## Material and methods

### Study design and study population

**Fig. 1:**
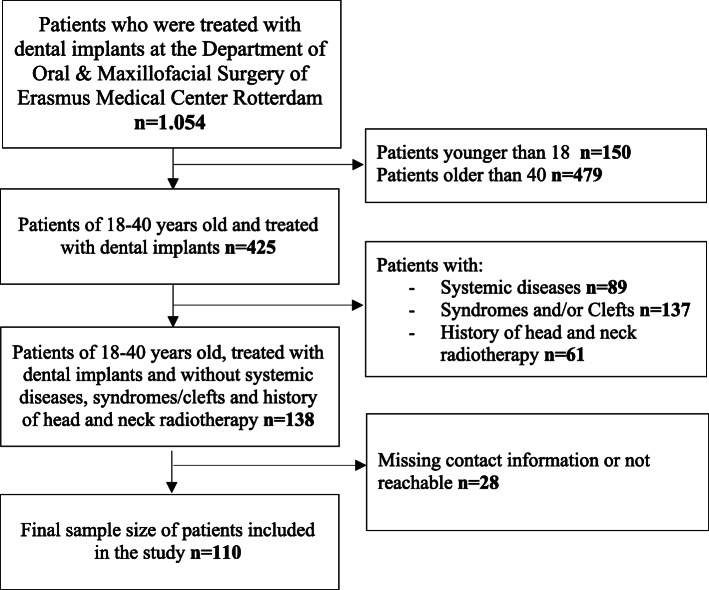
Flowchart of the study population

In this cross-sectional study, inclusion criteria were (1) patients who were diagnosed with tooth loss or tooth agenesis (hypodontia or oligodontia); (2) patients who were examined and treated with dental implants since 2006 at the Department of Oral & Maxillofacial Surgery of Erasmus Medical Center in Rotterdam, the Netherlands; (3) patients between 18 and 40 years old. As shown in the literature, a higher age is associated with a higher risk of implant loss [30, 31], this study only included patients of age < 40 years old in order to minimize the confounding effect of age on the clinical outcomes of DIT as much as possible. In total, 1054 patients were evaluated for the eligibility to participate in the study (Fig. 1). The following exclusion criteria were considered: (1) patients older than 40 years old (*N* = 479) and younger than 18 years old (*N* = 150); (2) presence of systemic diseases and history of head and neck radiotherapy (*N* = 150); (3) syndromic tooth agenesis (e.g., ectodermal dysplasia, clefts; *N* = 137); (4) patients with no contact records available or who were unreachable (*N* = 28). Therefore, the final study population consisted of 110 patients, of which 32 patients were diagnosed with tooth loss, 25 patients had hypodontia, and 53 patients were diagnosed with oligodontia. The medical records and dental panoramic radiographs (DPRs) of the patients were evaluated by an independent and trained examiner (Y. A.N.) from October until end of November 2018. The utilization of DPRs was performed in accordance with the general treatment protocol. The general information of the patients was used in strict compliance with the patient privacy regulation of the Erasmus Medical Center and consent of the patients. The study was conducted in accordance with the Declaration of Helsinki and was approved by the Medical Ethics Committee of the Erasmus Medical Center in Rotterdam, the Netherlands (MEC-2020-0301).

### Exposure assessment: the cause of missing teeth

Missing teeth were assessed during the clinical examination by dental professionals and were in addition determined in the DPRs or medical records of the patients. A tooth was classified as congenitally missing when no sign of formation or calcification was shown in the DPR taken between the ages of 6 and 15 years. Patients with 1 or more congenitally missing teeth, excluding third molars, were diagnosed with tooth agenesis and composed the group of patients with missing teeth due to congenital causes classified into the hypodontia (1–5 missing teeth) and oligodontia (6–27 missing teeth) group [1, 35]. Finally, patients who had a history of tooth extractions or tooth loss because of severe periodontitis, severe caries or dental trauma, were included in the group of patients with tooth loss. Patients with tooth loss were assigned as the reference group of the study.

### Surgical protocol

The surgical procedures were performed under local or general anesthesia. The position of the dental implant was determined by preoperative radiological measurements, dental setup, and surgical guide. Bone level implants with 3.3 and 4.1 mm diameter (Straumann® Bone Level, Basel, Switzerland) were inserted. Bone augmentation was performed in patients with insufficient bone volume, either as a separate pre-implantological procedure (4 months prior to implant placement) or at implant placement in case of relatively small defects with titanium dehiscence of less than 4 mm. The bone was harvested either from the ascending ramus or extra-orally from either the outer skull or the iliac crest. In addition, alloplastic material (Straumann Cerabone) was used in a mix with autogenous bone chips. In general, implant placement was performed in two stages with a period of 4 months in between [19].

### Assessment of implant outcomes: implant survival and subjective implant success

#### Implant survival

The primary outcome variable is the survival time of implants (years) and is defined as the time difference between the moment of the last contact/visit or implant loss and the date of first implantation. Implant loss is defined as failure of osseointegration.

#### Subjective implant success

The secondary outcome variable is the subjective implant success rate and is defined based on seven important implant outcomes (Table 1): (1) absence of mobility, (2) lack of persistent subjective complaints, (3) absence of recurrent peri-implantitis with suppuration, (4) absence of a continuous radiolucency around the implant, (5) pocket probing depth (PPD) not higher than 5mm, (6) no bleeding on probing (BOP), and (7) minimal bone resorption (less than 1.5 mm) observed in X-ray image [11].

**Table 1:** Criteria used to measure success rate of dental implants

Criterion	Used	Question asked
Absence of mobility [6]	Yes	Are the implants mobile?
Absence of persistent subjective complaints (pain, foreign body sensation and/or dysesthesia) [6]	Yes	Do you have any complaints in regard to the implants? Are you satisfied with the implants?
Absence of recurrent peri-implant infection with suppuration [6]	Yes	Are there any recurrent infections noticed by your dentist or oral hygienist?
Absence of a continuous radiolucency around the implant [6]	No	–
No pocket probing depth (PPD) > 5 mm [5, 28]	Yes	Is there any bleeding noticed when brushing or cleaning interdentally?
No PPD ≥ 5 mm and bleeding on probing (BOP) [28]	Yes	Is there any bleeding noticed when brushing or cleaning interdentally?
During the first year, a 1.5-mm vertical bone resorption was accepted. After the first year of service, the annual vertical bone loss should not exceed 0.2 mm (mesially or distally) ([2], Albrektsson and Isidor 1994)	No	–

In impossibility to achieve a control visit for all the patients and measure the objective and subjective implant outcomes, implant outcomes were recorded from an individual Dutch questionnaire with seven closed (‘yes’ or ‘no’) questions derived from the above-mentioned criteria. The eligible patients were reached by e-mail or phone. Patients were asked whether they (1) had lost the implant(s), (2) had noticed mobility of the suprastructure(s), (3) had complaints related to the implant(s), (4) were functionally and esthetically satisfied with the implant(s), (5) visited the dentist on a regular basis (twice a year) since the implant(s) was (were) placed, (6) had experienced recurrent infections around the implant(s) noticed by the dentist or oral hygienist, and (7) had noticed bleeding gums around the implant(s) while tooth-brushing or cleaning interdentally. Dichotomized variables on implant outcomes were created from the patients’ answers and were used as dependent variables in the following statistical analysis.

### Covariates

Information about general characteristics such as age, sex, ethnicity, and smoking were extracted from the clinical patient database (Chipsoft Healthcare Information X-change (HiX) program). Information about surgical treatment and treatment characteristics such as date of first implantation, number of dental implants, implant loss, number of missing teeth, need for bone augmentation, type of bone graft, morbidity, and additional surgical interventions was also collected from the patient medical records using HiX program.

### Statistical analyses

The study population was characterized using descriptive statistics. Differences in general characteristics and implant outcomes among patients with hypodontia, oligodontia, and tooth loss (reference group) were evaluated using Mann-Whitney non-parametric test for continuous variables with a skewed distribution and chi-squared test for categorical variables. Implant survival function was presented using the Kaplan-Meier analysis considering the implant loss events during the follow-up period. Log-rank tests were used to analyze differences in survival rates between the subgroups (hypodontia vs. tooth loss and oligodontia vs tooth loss). Differences in implant outcomes (implant loss, satisfaction, complaints, mobility, bleeding, and recurrent infections) between patients with tooth loss and patients with missing teeth due to congenital causes (hypodontia and oligodontia) were studied using binary logistic regression analysis, from which OR (odds ratios) and 95% CI were obtained from two consecutive models. In both models, the cause of missing teeth is considered as the main determinant and implant loss, patient satisfaction, complaints, implant mobility, bleeding, and recurrent infections as the primary outcomes. In model 1, the confounding effects of age, sex, smoking and number of missing teeth were taken into consideration. Subsequently, model 2 was additionally adjusted for bone augmentation and accompanying surgical procedures involving the craniofacial structures. The covariates were included in the logistic regression models based on previous literature or a change of > 10% in effect estimates. For all analyses, statistical significance was reached for p value < 0.05. All statistical analyses were performed using statistical package for social sciences SPSS version 24.0. At last, this study is in compliance with the STROBE checklist (Supplementary Table 1).

## Results

### Subject characteristics

The baseline characteristics of the study population are shown in Table 2.

**Table 2:** Baseline characteristics of the study population

	^*^ Non-congenital missing teeth (***N*** = 32)	Hypodontia (***N*** = 25)	*p* value	Oligodontia (***N*** = 53)	*p* value
**General characteristics**					
Age (years)	26.0 (21.7–38.0)	23.0 (18.3–27.7)	*0.007*	25.0 (19.4–29.6)	0.700
Sex (N, %)			*0.047*		0.983
Males	20 (62.5)	9 (36.0)		33 (62.3)	
Females	12 (37.5)	16 (64.0)		20 (37.7)	
Ethnicity (N, %)			0.680		0.144
Caucasians	26 (81.3)	22 (88.0)		48 (90.6)	
Africans	5 (15.6)	2 (8.0)		2 (3.8)	
Asian	1 (3.1)	1 (4.0)		3 (5.7)	
Smoking (N, %)			0.056		0.146
Yes	9 (28.1)	2 (8.0)		8 (15.1)	
No	23 (71.9)	23 (92.0)		45 (84.9)	
Missing teeth	3 (1–10)	3 (1–5)	0.855	9 (6–18)	*< 0.001*
Maxilla	2 (0–7)	2 (0–5)	0.879	5 (2–8)	*< 0.001*
Mandible	1 (0–7)	1 (0–4)	0.879	4 (1–10)	*0.002*
Missing teeth overall (N, %)			0.517		*< 0.001*
Maxilla	66 (55.5)	48 (64.0)	0.909	287 (53.5)	*< 0.001*
Mandible	54 (45.0)	27 (36.0)	0.798	249 (46.5)	*< 0.001*
Placed implants per patient					
Maxilla	2 (1–4)	1 (0–5)	0.855	3 (1–5)	*< 0.001*
Mandible	0 (0–4)	1 (0–3)	0.907	3 (0–6)	*0.004*
Placed implants overall (N, %)					
Maxilla	51 (60.7)	35 (63.6)	0.928	165 (54.5)	*0.002*
Mandible	33 (39.3)	20 (36.4)	0.325	138 (45.5)	*0.013*
Type of bone graft (N, %)			0.234		0.319
Ascending ramus	18 (36.7)	13 (52.0)		28 (52.8)	
Extra-oral	4 (8.2)	0 (0.0)		8 (15.1)	
Skull	1	-		3	
Iliac crest	3	-		5	
Alloplastic	1 (2.0)	1 (4.0)		0 (0.0)	
No bone augmentation	26 (53.1)	11 (44.0)		17 (32.1)	
Additional procedures (N, %)			0.650		0.176
BSSO	5 (15.6)	2 (8.0)		3 (5.7)	
SARME/Bimax	2 (6.2)	1 (4.0)		13 (24.5)	
Other surgical procedures	0 (0.0)	0 (0.0)		3 (5.7)	
No surgical procedures	25 (78.1)	22 (88.0)		34 (64.2)	
Regular visits at the dentist (N, %)			0.217		0.053
Yes	24 (75.0)	22 (88.0)		48 (90.6)	
No	8 (25.0)	3 (12.0)		5 (9.4)	
**Implants outcomes**					
Follow-up time (years; median)	3.3 (0.6–12.5)	2.7 (0.2–9.0)	0.342	4.2 (0.3–9.9)	0.301
Follow-up time (years; mean)	4.4 (3.1–5.6)	3.1 (2.1–4.1)	0.106	4.3 (3.5–5.1)	0.902
Lost implants (N, %)					
Maxilla	0 (0.0)	2 (5.7)	0.254	3 (1.8)	0.171
Mandible	1 (3.0)	0 (0.0)	0.373	3 (2.2)	0.593
Satisfaction (N, %)			*0.039*		*0.044*
Yes	27 (84.4)	25 (100.0)		51 (96.2)	
No	5 (15.6)	0 (0.0)		1 (1.9)	
N/A	0 (0.0)	0 (0.0)		1 (1.9)	
Complaints (N, %)			0.706		0.408
Yes	4 (12.5)	4 (16)		3 (5.7)	
No	28 (87.5)	21 (84)		49 (92.5)	
N/A	0 (0.0)	0 (0.0)		1 (1.9)	
Mobility (N, %)			0.203		0.714
Yes	2 (6.3)	0 (0.0)		4 (7.5)	
No	30 (93.8)	25 (100)		48 (90.6)	
N/A	0 (0.0)	0 (0.0)		1 (1.9)	
Bleeding (N, %)			0.452		0.645
Yes	16 (50.0)	10 (40.0)		29 (54.7)	
No	16 (50.0)	15 (60.0)		23 (43.4)	
N/A	0 (0.0)	0 (0.0)		1 (1.9)	
Recurrent infections (N, %)			0.479		0.460
Yes	9 (28.1)	5 (20.0)		20 (37.7)	
No	23 (71.9)	20 (80.0)		32 (60.4)	
N/A	0 (0.0)	0 (0.0)		1 (1.9)	

*Footnote*: *- reference group, *N* number of participants

Values are numbers and percentages for categorical variables or medians (90% range) for ordinal and continuous variables with a skewed distribution Follow-up time is presented as median (90% CI) and mean (95% CI). Differences were tested using the Mann-Whitney U non-parametric test for continuous variables and chi-squared test for categorical variables; *p < 0.05* is considered statistically significant and presented in italics

Patients included in the study were followed up for a median time of 3.6 years (90% CI, 0.4–10.2 years). The mean follow-up of patients in the oligodontia group was 4.3 years (95% CI, 3.5–5.1 years), in the hypodontia group 3.1 years (95% CI, 2.1–4.1 years), and in the tooth loss group 4.4 years (95% CI, 3.1–5.6 years). No statistically significant differences in both median and mean follow-up period were observed in patients with hypodontia and oligodontia compared to patients with tooth loss. A significantly higher number of maxillary and mandibular teeth were missing in patients with oligodontia (median = 9 teeth; 90% range, 6–18 teeth) than in patients with tooth loss (median = 3 teeth; 90% range, 1–10 teeth). In consequence, more implants per patient (maxilla *p < 0.001*; mandible *p = 0.004*) and more implants overall (maxilla *p = 0.002*; mandible *p = 0.013*) were placed in the oligodontia group than in the reference group. Compared to the patients with tooth loss, the patients with hypodontia showed no statistically significant difference in the number of missing teeth (p = 0.855) and the number of placed implants (p = 0.234). In comparison with the reference group, the hypodontia group had significantly younger participants (p = 0.007) and more female participants (*p = 0.047*). No statistically significant differences in ethnicity, smoking, bone augmentation, additional procedures, and regular visits at the dentist after DIT were shown in the hypodontia and oligodontia groups compared with the tooth loss group (p > 0.05).

### The cause of missing teeth and implant survival

**Fig. 2:**
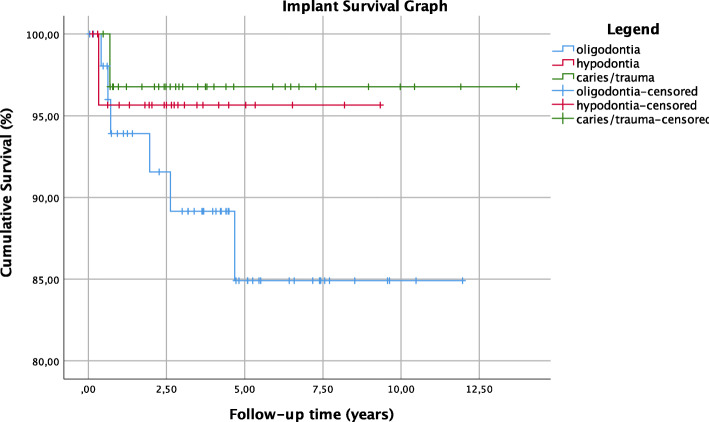
Presentation of the implant survival function. Footnote: the non-congenital tooth loss group of patients showed a cumulative implant survival rate of 96.9% (mean survival = 13.27 years; 95% CI 12.46, 14.08 years), the hypodontia group showed a cumulative implant survival rate of 96.0% (mean survival = 8.94 years; 95% CI 8.19, 9.70 years) and the oligodontia group showed a cumulative implant survival rate of 88.7% (mean survival = 10.50 years; 95% CI 9.40, 11.60 years)

One implant out of 84 had failed osseointegration in patients with tooth loss, providing a cumulative survival rate of 96.9% (mean survival = 13.27 years; 95% CI 12.46, 14.08 years). Two implants out of 55 were lost in patients with hypodontia, resulting in a cumulative survival rate of 96.0% (mean survival = 8.94 years; 95% CI 8.19, 9.70 years). Six implants out of 303 had failed in patients with oligodontia, resulting in a cumulative survival of 88.7% (mean survival = 10.50 years; 95% CI 9.40, 11.60 years) (Fig. 2). The log-rank test showed no statistically significant difference in the survival rate of implants among the groups of oligodontia vs. hypodontia (p = 0.425), oligodontia vs. tooth loss (p = 0.210) and hypodontia vs. tooth loss (p = 0.785). The median survival time of the failed implants was 1.3 years in the oligodontia group, 0.3 years in the hypodontia group, and 0.7 years in the tooth loss group.

### The cause of missing teeth and subjective implant success

**Fig. 3:**
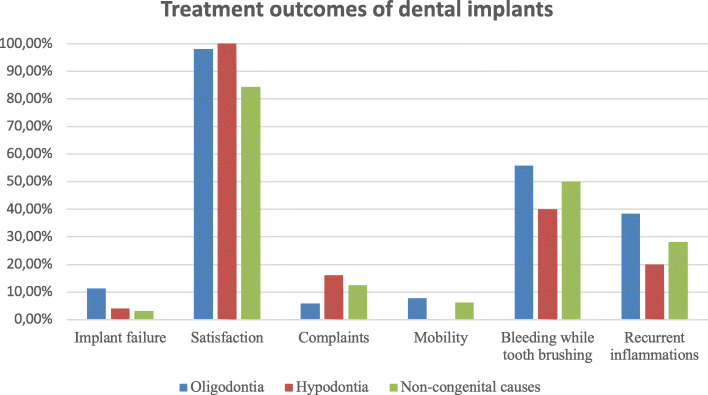
Presentation of the subjective implant success. Footnote: y-axis presents the % of patients for each respective outcome between the three groups: lost implants (*p* = 0.101); satisfied patients (*p=0.037*); presence of complaints (*p* = 0.505); mobility of implants (*p* = 0.547); bleeding noticed during tooth brushing (*p* = 0.597); presence of recurrent inflammation around the implant (*p* = 0.410)

As shown in Table 3 and Fig. 3 the cause of missing teeth (congenital vs non-congenital) was not statistically significantly associated with failure of osseointegration, complaints, mobility, bleeding, and peri-implantitis, when considering for potential confounders in both models 1 and 2. In model 1, patients with congenitally missing teeth (hypodontia and oligodontia) were significantly more satisfied with the dental implants compared to patients with tooth loss (OR = 19.71, 95% CI 1.33, 292.19). Patient satisfaction remained significantly higher (*p < 0.040*) in patients with congenital missing teeth in model 2; however, the effect estimate decreased (OR = 18.18, 95% CI 1.14, 289.15) (Fig. 3).

**Table 3:** Association between the cause of missing teeth and implants outcomes

Congenital vs non-congenital causes	Model 1			Model 2		
	OR	95% CI	*p* value	OR	95% CI	*p* value
Implant loss	5.25	0.25, 108.33	0.283	4.17	0.24, 71.72	0.326
Satisfaction	19.71	1.33, 292.19	0.030*	18.18	1.14, 289.15	0.040*
Complaints	0.55	0.12, 2.49	0.435	0.70	0.14, 3.49	0.667
Mobility	0.84	0.07, 9.48	0.886	0.88	0.08, 9.21	0.912
Bleeding	1.12	0.40, 3.18	0.827	1.07	0.37, 3.07	0.905
Recurrent inflammations	1.43	0.44, 4.70	0.555	1.47	0.45, 4.88	0.525

*Footnote*: *OR* odds ratio; *statistically significant p values are presented in italics

Model 1 was adjusted for age, sex, smoking (yes or no), and number of missing teeth (continuous)

Model 2 was additionally adjusted for bone augmentation (yes or no) and additional surgical procedures involving craniofacial structures (yes or no)

## Discussion

Findings from this cross-sectional study with retrospective measurement of exposure, suggest that the cause of missing teeth does not influence implant survival. Furthermore, the cause of missing teeth has no significant impact on implant success components naming failure of osseointegration, complaints, implant mobility, bleeding, and peri-implantitis. Interestingly, the only component of implant success influenced by the cause of missing teeth is patient satisfaction. Young patients with congenitally missing teeth are significantly more satisfied with their implants compared to patients with missing teeth due to caries, periodontitis or trauma.

### Interpretation of the main findings

The evaluation of the cause of missing teeth as an indicator of implant survival and success is unraveled in the literature. In this study, the relationship between cause of missing teeth and implant outcomes was hypothesized based on existing evidence about decreased bone quantity and quality in patients with congenital missing teeth [32]. In addition, tooth agenesis indicates often aberrant occlusal traits mentioning the tendency towards class III malocclusion, which can highly influence the longevity of dental implants in jaws [8]. Based on these facts, a lower survival and success rate of implants in patients with congenital missing teeth was expected, but not shown in the findings of our study.

#### Implant survival

Literature has shown lower implant survival rates in patients with congenitally missing teeth in comparison to patients with tooth loss [7, 14, 20, 21, 23–26]. In a short-term follow-up period, comparative results were found, showing a cumulative survival rate of 96.9% for patients with tooth loss, 96.0% for patients with hypodontia, and 88.7% for patients with oligodontia. Although, a statistically significant difference in DIT survival could not be proved, a deceleration trend of implant survival was observed in ascending order in patients with tooth loss, hypodontia, and oligodontia. Furthermore, the implant survival tends to decelerate with the increase in number of missing teeth. Patients with oligodontia are characterized by more missing teeth in comparison to patients with hypodontia or patients with tooth loss. More missing teeth are accompanied by the need of placing more implants to recover the function of the dentition. On the other hand, more missing teeth are implicated in the decrease of cortical bone density, which thoroughly compromises the survival and the success of dental implants [32].

#### Subjective implant success

Literature has reported lower implant success rates regarding DIT in patients with tooth loss, in comparison to patients with congenital missing teeth [27]. Most of the studies on dental implants use the term success rate synonymously with the term survival rate, which leads to biased interpretation and overestimations of success rates, which are usually lower than the survival rate of implants. Therefore, difficulties were faced in selecting reported values in the literature for the interpretation of our findings about the subjective implant success based on the DIT outcomes (including patient satisfaction, complaints, implant mobility, bleeding, and recurrent inflammations). The systematic review of Filius et al. reported very high satisfaction rates (> 85%) for DIT in patients diagnosed with hypodontia and oligodontia [14]. Topcu et al. studied satisfaction of patients diagnosed with tooth loss and reported a satisfaction rate of 87.4% (varying from 71% to 99%) [38]. In line with these results, significantly high satisfaction rates of patients with hypodontia (100%) and patients with oligodontia (98%) were demonstrated, compared to patients with tooth loss (84.4%). Topcu et al. reported that improved satisfaction is related to improved esthetics and function in comparison to the old oral state [38]. In general, patients with congenital missing teeth undergo a long and intense dental treatment of several disciplines, restorative dentistry, orthodontics, prosthodontics, and oral and maxillofacial surgery. This group of patients tend to compare the function and esthetics of the current dentition restored by dental implants with the past status of the dentition predominated by unaccomplished esthetics due to absence of several teeth and presence of malformed teeth. Therefore, patients with tooth agenesis are prone to feel more satisfied with their implants since the improvement of function and esthetics of their dentition is highlighted after restoration. Whereas, patients with tooth loss are likely to feel less satisfied with dental implants, because they tend to compare the current status of the dentition with their past complete and healthy dentition, prior experiencing tooth loss due to caries, periodontitis and trauma.

Further, Lima et al. reported that patient’s expectation prior to DIT is an important factor for the patient satisfaction of DIT [10]. As stated before, patients who are diagnosed with tooth agenesis follow long and intense treatments being in continuous contact with the clinicians from the moment of diagnoses. Meanwhile, patients with tooth loss are in general less frequently in contact with the clinicians. Therefore, patients with tooth agenesis have a clearer perception of their situation and a more realistic expectation of DIT, which also contributes to a higher patient satisfaction of DIT of patients with tooth agenesis.

Previous literature reported that in patients with oligodontia the bleeding on probing (BoP) rate is 32% [17]. Farina et al. reported BoP rates of 27% around implants of patients with tooth loss [13]. In this study, bleeding and recurrent inflammations tended to occur more frequently in patients with oligodontia (55.8% and 38.5% respectively) than in patients with hypodontia (40.0% and 20.0%, respectively) or in patients with tooth loss (50% and 28.1%). However, no statistical significance could be proven on the effect of the cause of missing teeth on the subjective success of implants considering bleeding and recurrent inflammations.

### Strengths and limitations

For the interpretation of the study results, some strengths and limitations have to be considered.

As the cause of missing teeth has not been previously considered as a determinant of implant outcomes, the findings of this study add knowledge to the current literature about the factors that influence implant survival and success. The cause of missing teeth was evaluated as an indicator of implant outcomes by providing scientific evidence from a clinical retrospective observation. One of the strengths of the current study is the emphasis given to the subjective evaluation of satisfaction and complaints as implant outcomes. Both representing important components of implant success which are often overshadowed in the literature by the high reported survival rates [4, 7, 9, 15, 18, 25, 39]. The population size in the present study was relatively small which leads to decreased statistical power and might also have affected the significance of the effect estimates and p values. The retrospective nature of the study design counts as another limitation because it can generate selection and recall bias. Consequently, the control on the outcome assessment might have been affected. In addition, retrospective designs require very large sample sizes to study such rare outcomes. Therefore, it is important to underline the need of a large-scale dataset and a long term follow-up to assess implant survival and success for the performance of future studies. Tooth agenesis, however, can be considered as a rare congenital dental anomaly and quite challenging to achieve large sample size for the study population.

Bleeding and recurrent inflammations were assessed subjectively by asking the patients questions via call or mail. Although inapplicable for the current study, the best way to assess bleeding and recurrent inflammations would be to continuously follow-up the patients by organizing control examinations. Moreover, radiolucency around the implant and annual peri-implant bone loss (≤ 0.2 mm) 1 year after insertion of the superstructure [11] was not assessed because in most of the cases a recent radiograph was not present. The criteria of Donos et al. were applied to define implant success, because the criteria included by the authors considered all implant success definitions of each study included in their systematic review [11]. However, a lack of consensus or clear definition of implant success is present in the literature. There is a high need for a unified score system where patient satisfaction and implant survival are combined to define implant success rate.

In conclusion, the cause of missing teeth does not affect the short-term survival of dental implants but it does indicate the subjective implant success from the perspective of patient satisfaction. Patients with tooth agenesis and with an increased number of missing teeth are more content about the treatment with dental implants than patients with tooth loss. A consensus in the literature regarding the assessment of implant success is an essential concern for clarification by future research.

## Supplementary Information

**Additional file 1: Supplementary Table 1**. STROBE Statement.

## Data Availability

Supporting data is available.

## Abbreviations

DIT: Dental implant treatment
DPR: Dental panoramic radiographs
HiX: Chipsoft Healthcare Information X-change program
PPD: Pocket probing depth
OR: Odds ratio
CI: Confidence interval
